# Neonatal weight loss and gain patterns in caesarean section born infants: integrative systematic review

**DOI:** 10.1111/mcn.12914

**Published:** 2019-11-27

**Authors:** Niamh M. Kelly, Jessica V. Keane, Rachel B. Gallimore, Debra Bick, Rachel M. Tribe

**Affiliations:** ^1^ Dept. of Women and Children's Health, School of Life Course Sciences, Faculty of Life Sciences and Medicine, St Thomas' Hospital Campus King's College London London UK; ^2^ Maternity Department Imperial College Healthcare NHS Trust London UK; ^3^ Warwick Clinical Trials Unit, Warwick Medical School University of Warwick Coventry UK

**Keywords:** breastfeeding, caesarean section, excessive weight loss, infant feeding, infant growth, mode of delivery, neonatal growth, neonatal weight loss

## Abstract

There is evidence that caesarean section delivery can impact on neonatal weight loss and weight gain patterns in the first 5 days of life. We conducted an integrative systematic review to examine the association of mode of delivery on early neonatal weight loss. Pubmed, Cumulative Index to Nursing and Allied Health Literature, Web of Science, Excerpta Medica dataBASE, and Medical Literature Analysis and Retrieval System Online were searched for relevant papers published before June 2019. Reference lists from the relevant papers were then backwards and forwards searched. As neonatal weight loss was reported in different formats, a meta‐analysis could not be carried out. Most studies did not distinguish between elective and emergency caesarean sections or instrumental and nonassisted vaginal deliveries. Seven papers were included. All papers except one found that caesarean section was associated with higher weight loss in the early days of life. Two papers presented data from studies on babies followed up to 1 month. One study found that on day 25, babies born by caesarean section had significantly higher weight gain than those born vaginally, while another found that by day 28, babies born vaginally gained more weight per day (11.9 g/kg/day) than those born by caesarean section (10.9 g/kg/day; *p* = .02). Overall, infants born by caesarean section lost more weight than those born vaginally, but due to the small number of studies included, more are needed to look at this difference and why it may occur. This discrepancy in weight between the two groups may be corrected over time, but future studies will need larger sample sizes and longer follow‐up periods to examine this.

**Key messages**

- Infants born by caesarean section may be at a higher risk of increased weight loss.
- It is not clear what factors contribute to increased weight loss in these infants.
- It is not known to what extent this weight loss is detrimental to the infant.
- Enhanced feeding support may be needed for babies more at risk of weight loss.
- More awareness for clinicians may be beneficial in further informing and supporting families.

## 1 INTRODUCTION

Most babies are routinely weighed following birth and again within the first week of life (World Health Organization, 2015). It is common and physiologically normal for babies to lose a small amount of weight within the first few days of birth due to body fluid adjustments [Modi, Bétrémieux, Midgley, & Hartnoll, 2000; National Institute for Health and Care Excellence (NICE), 2017]. However, guidelines state that infants usually then surpass their birthweight by around day 14 (Department of Health, 2009a, 2009b). World Health Organization suggests a loss of 10% of birthweight in the first week of life as a threshold for further assessment (World Health Organization, 2015), with the UK NICE recommending a clinical examination to exclude dehydration, obtaining a feeding history and an observation of a feed if weight loss exceeds this (NICE, 2017). The American Academy of Pediatrics suggest that >7% weight loss within the first 3 to 5 days is excessive for a healthy, full‐term, breastfed infant (American Academy of Pediatrics & The American College of Obstetricians and Gynecologists, 2012). It is unclear what the evidence is behind these thresholds, with various limits described as “excessive weight loss” in healthy full‐term infants ranging from 7% up to 12.5% of infant birthweight (Chantry, Nommsen‐Rivers, Peerson, Cohen, & Dewey, 2011; Davanzo, Cannioto, Ronfani, Monasta, & Demarini, 2013; Mulder & Gardner, 2015). As this classification of excess weight loss varies from country to country, it is difficult to know the true prevalence. NICE acknowledges the need for robust evidence to examine routine weighing of babies in the first 6–8 weeks, the importance of feed intervals, and the management of faltering weight (NICE, 2015, 2017).

Regardless of variation in threshold, it is important to identify and act appropriately in response to excess infant weight loss as it is associated with serious health outcomes including hyperbilirubinaemia, hypernatremic dehydration, and failure to thrive (Van Dommelen, Van Wouwe, Breuning‐Boers, Van Buuren, & Verkerk, 2007). Birth by caesarean section (CS) is a risk factor for excess neonatal weight loss, with several reasons for this proposed including physiological, mechanical, and environmental problems: excess mucous in the infant's respiratory tract, delayed lactogenesis, delayed time of feeding initiation, increased load of IV fluids women receive during CS, maternal post‐operative pain, maternal impaired mobility, and separation of infant due to neonatal unit admission (Awi & Alikor, 2006; Chantry et al., 2011; Dewey, Nommsen‐Rivers, Heinig, & Cohen, 2003). Successful feeding is essential to prevent excessive weight loss, but this can be impacted further if maternal CS complications arise such as emotional distress, particularly if the CS was unexpected, and post‐operative pain (Carlander, Edman, Christensson, Andolf, & Wiklund, 2010; Karlström, Engström‐Olofsson, Norbergh, Sjöling, & Hildingsson, 2007). The reason for CS, and whether it was elective or emergency, may also influence these factors and subsequent infant weight loss. However, as yet, studies have not been conducted to consider all of these elements and adjust for any confounding variables.

Other factors that may contribute to infant weight loss in the first few days and weeks post‐birth include feeding method and epidural analgesia in labour (Martens & Romphf, 2007), which may not be considered in antenatal advice offered to women. Infant gender has also been linked to differences in weight trajectory (Department of Health, 2009a, 2009b), with female infants losing more weight in the early days of life than males (Martens & Romphf, 2007). Advice provided by the UK National Health Service does not acknowledge mode of birth as influencing infant weight loss (National Health Service, 2017). Current Royal College of Obstetricians and Gynaecologists' guidelines on CS consent do not include any recommendations that risk of increased infant weight loss should be discussed with women planning a CS birth (Royal College of Obstetricians and Gynaecologists, 2009). Further research is needed to determine whether infant weight loss relating to these factors is detrimental to the infant's health and whether interventions are necessary or beneficial.

Management plans for infants with excess weight loss generally focus on promoting weight gain but do not always focus on promotion of consistent evidence‐based infant feeding support to parents. Formula supplementation is very often part of the management plan for exclusively breastfed babies. NICE (2017) acknowledges that formula supplementation in breastfed infants experiencing excessive weight loss may support weight gain; but United Nations Children's Fund suggests that offering formula supplementation is likely to have a detrimental effect on breastfeeding (UNICEF, 2019) including if this is against the wishes of the woman. This can also lead to longer term health implications linked to formula feeding including increased risk of gastrointestinal and respiratory infections (Duijts, Ramadhani, & Moll, 2009; Gale et al., 2012) and impact on breastfeeding duration (Brown, 2015). It is also likely to increase feelings of stress, concern, and anxiety for parents or caregivers (Flaherman, Beiler, Cabana, & Paul, 2016). If excess infant weight loss persists, management plans could require hospital admission or emergency department attendance.

The purpose of this systematic review was to examine the evidence that infants born by CS have a higher weight loss in the early days of life than those born vaginally and comment on potential reasons for this. Our secondary aim was to assess whether weight disparities are significant beyond the initial weight loss period.

## 2 METHODS

Different approaches to review of the literature were considered (Aveyard & Bradbury‐Jones, 2019). An integrative approach was deemed the most appropriate for this systematic review due to the variation in study methods and how weight loss information was presented. The integrative systematic review was conducted according to the five‐step framework described by Whittemore and Knafl (2005), that is, (a) problem identification, (b) literature search, (c) data evaluation, (d) data analysis, and (e) presentation, and according to the Preferred Reporting Items for Systematic Reviews and Meta‐Analyses' guidelines. It was registered with PROSPERO, the International Prospective Register of Systematic Reviews, University of York; CRD42019125908.

### 2.1 Literature search

**Figure 1:**
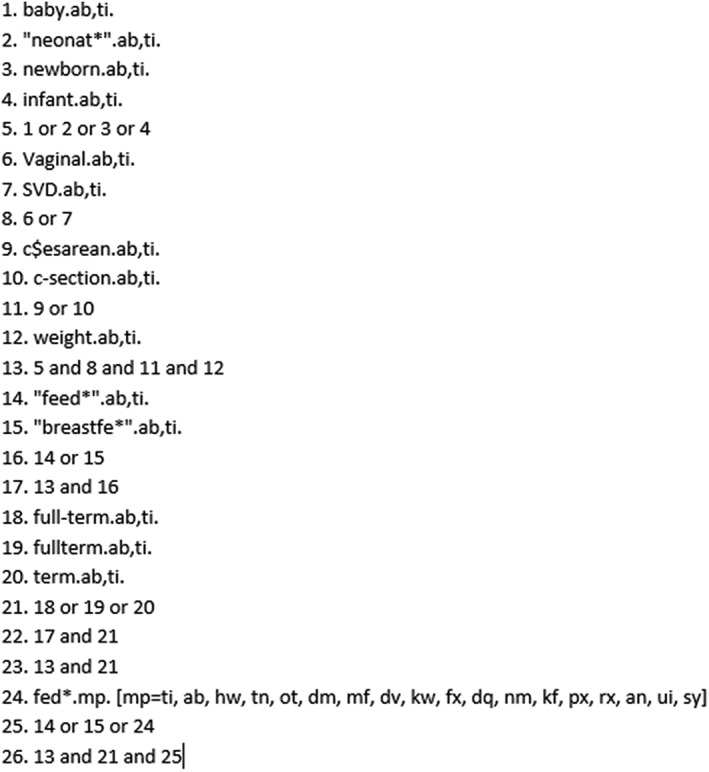
Electronic search strategy (Medical Literature Analysis and Retrieval System Online and Excerpta Medica dataBASE)

Papers were identified using PubMed, Medical Literature Analysis and Retrieval System Online, Excerpta Medica dataBASE, Cumulative Index to Nursing and Allied Health Literature, and Web of Science databases by NK and JK separately. Titles and abstracts of identified studies were independently screened by two authors, NK and RG. PubMed was searched using the following terms (((((((baby) OR newborn) OR neonat*)) AND vaginal) AND weight) AND (((caesarean) OR cesarean) OR c‐section)) AND (((fed*) OR feed*) OR breastfe*). Cumulative Index to Nursing and Allied Health Literature was also searched using the following terms; TX ( TX baby OR TX newborn OR TX neonat*) AND ( TX vaginal) AND ( TX weight) AND ( TX c#esarean ORTX c‐section) AND ( TX fed* OR TX feed* OR TX breastfe*). Web of Science was search with the terms (baby OR newborn OR neonat*) AND (vaginal) AND (weight) AND (c$esarean OR c‐section) AND (fed* OR feed* OR breastfe*)). The search terms used for Ovid (Medical Literature Analysis and Retrieval System Online and Excerpta Medica dataBASE) are shown in Figure 1.

Results were screened for inclusion according to the following criteria:

- healthy, full‐term singleton births;
- infant weight loss recorded within first 5 days of life;
- weight loss analysed based on mode of birth; and
- infants fed by the same method, for example, exclusively breastfed or exclusively formula fed.

No limits on type of study, country, or year of publication were applied due to the small number of studies fitting the above criteria.

### 2.2 Data evaluation

Full texts were assessed for relevance and evaluated for quality with the appropriate using the relevant Critical Appraisal Skills Programme (CASP) checklists for study design (NK, RG, and JK). A separate formal risk of bias assessment was not undertaken, as CASP checklists consider whether exposure and outcomes were adequately measured to reduce bias. The extent to which study authors identified potential confounding factors and took these into account in study design and analysis was also assessed (see Table 1).

**Table 1:** Summary of studies included in review

Author	Study methodology	Weight loss timepoint	Outcome	Critical appraisal score^a^	Limitations
Flaherman et al., 2015	Retrospective, 108,907 EBF infants; VD: 76.6%, CS: 23.4%	48–72 hr (discharge)	VD: median weight loss was 4.2, 7.1, and 6.4% at 24, 48, and 72 hr of age; by 48 hr, almost 5% had lost >10%. CS: median weight loss was 4.9, 8.0, 8.6, and 5.8% at 24, 48, 72, and 96 hr after delivery; by 48 hr, almost 10% had lost >10%.	8/12 Yes 2/12 Can't tell 2/12 No	Feeding reports obtained from medical records, weight measured at discharge, CS babies followed up for longer, does not report *p*‐value
Miller et al., 2015	Retrospective; 7,075 exclusively formula fed infants; VD: 64%, CS: 34%	48 and 72 hr	VD: median weight loss was 2.9% at 48 hr of age. By 48 hr, <5% of VD infants lost least 7% of their birth weight. CS: median weight loss at 48 and 72 hr were 3.7 and 3.5%, respectively; weight loss >8% occurred in <5% of infants.	6/12 Yes 4/12 Can't tell 2/12 No	Feeding reports obtained from medical records, sample may have included infants with comorbidities, does not report *p*‐value
Saki et al., 2010	Prospective; 92 EBF infants; VD: 62, CS: 38%	Three occasions; median of days 5, 15, and 30	Association between type of delivery and weight gain parameter estimate: 14.4, standard error: 5.4, *p* = .01 Boys VD: mean birth weight, 3,275.2 g (442.4 g), mean weight at day 5, 3,238.9 g (436.4 g) Boy CS: mean birth weight, 3,133.2 g (537.0 g), mean weight at day 5, 3,075.0 g (527.1 g) Girls VD: mean birth weight, 3,000 g (384.6g ), mean weight at day 5, 3,013.3 g (368.1g) Girls CS: mean birth weight, 3,165.4 g (265.7g), mean weight at day 5, 3,096.2 g (291.9g) Data for second and third weighing occasion published in Saki et al. (2010)	10/12 Yes 1/12 Can't tell 1/12 No	Infants were not weighed on the same day at each occasion, does not report percentage mean weight loss
Samayam et al., 2016	Prospective; 104 EBF infants, 47% VD, 53% CS	24 and 72 hr	Mean weight loss at 24 hr; CS: 3.2 % (1.9%) vs. VD: 2.2% (1.2%); *p* = .0016; Mean weight loss at 72 hr; CS: 5.9% (3.1%) vs. VD: 4.7% (2.5%); *p* = .0314; and Day 28 weight gain; CS: 10.9 g/kg/day (2.1 g/kg/day) vs. VD: 11.9 g/kg/day (2.3 g/kg/day); *p* = .0244	10/12 Yes 2/12 Can't tell 0/12 No	Small cohort, recruited over 3‐month period
Verd et al., 2018	Secondary analysis of prospective cohort study; 788 infants; initiated breastfeeding; 80% VD, 20% CS	Discharge—exact timepoint not recorded	Overall median of weight loss at discharge = 6%; % infants with weight loss <median: nonassisted 51%, instrumental 15%, and CS 34%; and % infants with weight >median: nonassisted 56%, instrumental 16%, and CS 28%; *p* = .21	8/12 Yes 2/12 Can't tell 2/12 No	Feeding reports obtained from medical records, weight loss recorded at discharge, weight loss refers to different days for mode of birth
Manganaro et al., 2001	Prospective cohort; 686 EBF infants Does not report breakdown of VD and CS	Maximal weight loss: VD, days 3–4; CS, days 4–5	77% of infants with weight loss >10% were born by CS, 36% of infants with weight loss <10% were born by CS; *p* < .001	8/12 Yes 1/12 Can't tell 3/12 No	Does not give exact day of weights. If infants were supplemented, does not state when or with what (formula/EBM)
Mezzacappa & Ferreira, 2016	Secondary analysis of prospective cohort of 414 EBF infants, 69% VD, 31% CS	Discharge—48 hr (VD), 72 hr (CS)	>8% weight loss 50.4% (n = 54) were born by CS ≤8% weight loss 24.1% (n=74) were born by CS *p* ≤ .0001, RR: 2.16	7/12 Yes 0/12 Can't tell 5/12 No	Weight loss measured at discharge, babies discharged >96 hr not included, variables associated with weight loss and breastfeeding difficulties not looked at

Abbreviations: CS, caesarean section; EBF, exclusively breastfed; EBM, expressed breastmilk; RR, relative risk; VD, vaginal delivery.

a The Critical Appraisal Skills Programme Cohort checklist was used to appraise each study.

### 2.3 Data analysis

As recommended for integrative reviews, we identified subgroup classifications and categorized our results by method of feeding, how neonatal weight loss was reported, and whether any tests for statistical significance were carried out (Whittemore & Knafl, 2005). A meta‐analysis was not undertaken because of variation in how data were reported.

## 3 RESULTS

**Figure 2:**
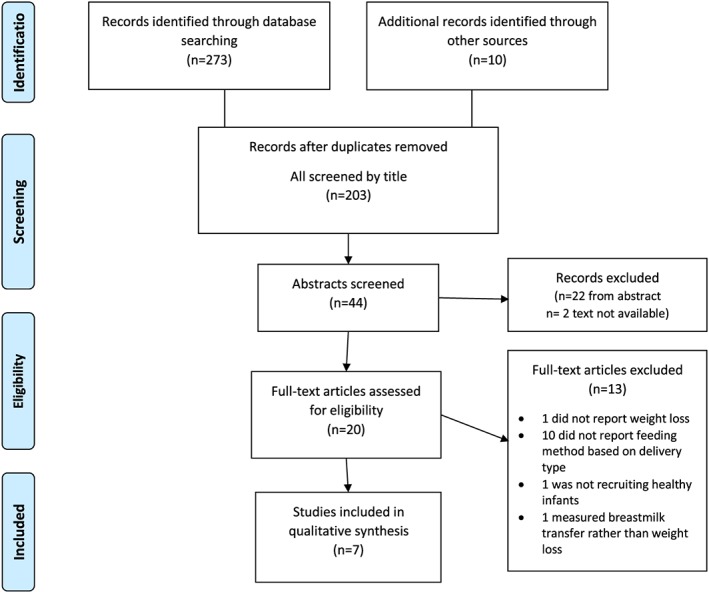
Flowchart of search strategy

The initial search yielded 273 papers, and hand searches of reference lists an additional three papers. Seven further papers were found by forward searching the papers identified. After removing duplicates and screening titles and abstracts, 20 papers remained. Ten papers did not report feeding method based on mode of birth and were omitted. Of the three papers excluded after reading the full text, one paper used infants' weights to create a model for prediction of weight loss but did not report actual weight loss, another measured breastmilk transfer rather than weight loss, and the third did not recruit healthy, full‐term singleton infants. This left seven papers for inclusion in the review (Figure 2).

Of the seven papers included, two were retrospective cohort studies (Flaherman et al., 2015; Miller et al., 2015), three were prospective cohort studies (Manganaro, Mamì, Marrone, Marseglia, & Gemelli, 2001; Saki, Eshraghian, Mohammad, Foroushani, & Bordbar, 2010; Samayam, Ranganathan, & Balasundaram, 2016), and two were secondary analyses of prospective cohorts (Mezzacappa & Ferreira, 2016; Verd, de Sotto, Fernandez, & Gutierrez, 2018). Two studies were carried out with the same United States of America (USA) cohort, and single studies were based in Italy, Spain, Iran, India, and Brazil. Study sample sizes varied from 92 to 108,907 infants. One paper assessed weight loss by mode of birth in exclusively formula fed infants (Miller et al., 2015), while the other six focused on breastfed babies. All studies recorded infant weight loss in the first 5 days of life, but only two followed infants for the first 4 weeks of life (Saki et al., 2010; Samayam et al., 2016). Unless stated, papers did not distinguish between emergency and elective CS or between instrumental and spontaneous vaginal birth. Using the CASP checklist for cohort studies, each paper was scored out of a maximum of 12 (Table 1).

A retrospective cohort study of 161,471 infants born in Northern California Kaiser Permanente hospitals, USA from 2009 to 2013 aimed to examine neonatal weight loss within the first few days of life (Flaherman et al., 2015). The authors analysed a subset of the cohort including all exclusively breastfed babies, with data extracted from the charts of 108,907 infants and weight loss nomograms created by mode of birth. A quantile regression model was developed to create percentiles of weight loss. As infants were weighed once daily in hospital, weight loss of babies born vaginally was determined from 6 to 72 hr following the birth; CS babies were weighed up until 96‐hr post‐birth due to longer inpatient hospital stays. The median percentage weight loss of babies born vaginally was 4.2, 7.1, and 6.4% at 24, 48, and 72 hr of age, respectively, while the median weight loss for babies born by CS was 4.9, 8.0, 8.6, and 5.8% at 24, 48, 72, and 96 hr after delivery (no ranges given). By 48 hr, almost 5% of babies delivered vaginally had lost >10% of their birthweight, while almost 10% of CS babies had lost >10% at the same timepoint. The authors do not report *p*‐values or comment on the statistical significance of these results.

To examine whether excluding babies once formula feeds were introduced led to selection bias and underestimated weight loss; the same researchers conducted a sensitivity analysis matching each censored infant with an uncensored infant (Flaherman et al., 2015). Weight data from the uncensored infant were used for the censored infant from the point when formula was introduced. Using these data, the authors repeated their analysis and found that the percentile curves from original data and imputed data were similar, thus the exclusion of babies once formula was introduced did not affect results. Any other confounding factors that may have been included in analysis were not described.

In contrast, Miller et al. (2015) analysed weight loss in the 7,075 exclusively formula fed infants in the same cohort. Median weight loss at 48 hr of age was 2.9% for babies delivered vaginally and 3.7 and 3.5% at 48 and 72 hr, respectively, for babies delivered by CS. By 48 hr, <5% infants lost at least 7% of their birthweight in the vaginally delivered group and >8% occurred in the CS group although *p*‐values or ranges for this analysis were not presented. A similar sensitivity analysis to that described above was carried out which also found that percentile curves of imputed data were similar to those of the original data.

In 2010, Saki et al., in a study from Iran, examined weight gain patterns of 92 exclusively breastfed infants. Women who attended health care centres in the city of Shiraz between 3 and 7 days post‐partum were recruited. Infants were weighed on three occasions: between days 3–7, 10–21, and 24–31 post‐partum, with median timepoints of 5, 15, and 30 days. Using Generalized Estimating Equation modelling, neonatal weight gain was significantly associated with mode of birth at all three timepoints [parameter estimate = 14.4 (5.4), *p* = .01]. Although babies born by CS lost more weight in the early days following birth and took longer to regain birthweight than babies born vaginally, the rate of weight gain increased in CS babies; by day 25, they had gained significantly more weight per day than those born vaginally. It is important to note that while more women who gave birth by CS received support with breastfeeding (during days 3–7), this was not significantly associated with increased weight gain in the first month of life. There was no association between neonatal weight loss and parity, maternal education, or infant gender (Saki et al., 2010).

Similarly, a prospective observational study analysed weight patterns during the first month of life in 104 exclusively breastfed infants born in a rural tertiary hospital in Bangalore, India, from August to October 2012 (Samayam et al., 2016). Fifty‐five babies (52.9%) were born vaginally and 49 (47.1%) by CS. Infants were weighed at birth and again on days 1, 3, 7, and 28. Unpaired *t*‐tests found that infants born by CS had a greater mean weight loss than those born vaginally both at 24 hr (3.2 and 2.2%, respectively, *p* = .0016) and at 72 hr (5.9 and 4.7%, respectively, *p* = .0314). By day 28, babies born vaginally had a higher mean weight gain (11.9 g/kg/day) than those born by CS (10.9 g/kg/day, *p* = .02). The study included no clinical information as to why the babies were born by CS or whether women had any medical or pregnancy complications. No details were included on what the maximum weight loss was, if any babies experienced excessive weight loss and if any babies received formula milk supplementation.

In the study by Manganaro et al. (2001), the primary focus was incidence of dehydration and hypernatremia in exclusively breastfed infants born at the Obstetric Clinic of University of Messina, Italy. For the purpose of the current review, only data pertaining to weight loss were included. CS was significantly associated with increased infant weight loss compared with those born vaginally (*p* < .001). Out of 686 healthy term infants, 53 lost >10% of their birthweight, with 77% of these infants born via CS. Of the infants who lost <10%, 36% were born via CS. Peak weight loss was recorded on day 4, on average showing vaginally born infants to have lost most weight on days 3–4 and CS infants on days 4–5. Following blood tests and feeding or residual milk assessments, 14 of these 53 infants were fed with formulated milk (11 born by CS); the remaining 39 continued breastfeeding with additional maternal support. By day 10, all babies had ‘normal growth', although the authors did not report how this was defined. All babies who initially had weight loss <10% and 39 (74%) of babies who lost >10% were still exclusively breastfeeding (*p* = .001) at 10‐days old.

Mezzacappa and Ferreira (2016) studied 414 full‐term exclusively breastfed babies from a baby friendly hospital in Brazil to identify factors involved in infants losing >8% of their birthweight at the time of hospital discharge. In this cohort, 286 (69%) of infants were born vaginally, and 128 (31%) were born by CS. The only significant factor found was CS birth. Birth by CS was associated with an increased risk of excess weight loss with 50.4% of those who lost >8% of birthweight being born by CS, while only 24% of those without excess weight loss were born by CS (adjusted relative risk: 2.27, 95% confidence interval [1.54–3.35]; *p* < .001), analysed using adjusted multiple and univariate Cox regression. There was wide variation in hospital length of stay between babies who lost <8% and >8% (58.0 ± 9.8 hr and 61.4 ± 9.9 hr, respectively, *p* = .003). This may be due to differences in inpatient discharge times among babies born vaginally and those born by CS. Although this was addressed and adjusted for in the regression analysis, other potential reasons for longer inpatient stay were not considered.

A prospective cohort of 788 exclusively breastfed mother‐baby dyads were recruited in Majorca, Spain to examine breastfeeding outcomes in women with mild gestational hyperglycaemia (Verd et al., 2018). The research team completed a secondary analysis to look at the link between breastfeeding and weight loss at discharge from hospital. Women were recruited at the first well‐baby visit to the paediatric clinic, with a total of 154 babies born by CS (19.5%). Babies were weighed at birth, every day until and including discharge from hospital, days 7, 15, 30, and 100, and analysed in two groups: ≤median weight loss and > median weight loss. Median weight loss was 6% and was not significantly different between delivery type (nonassisted vaginal delivery, instrumental, and CS) for either weight loss groups using chi‐square test, (*p* = .21). While Verd et al. did not mention whether babies were put on feeding plans if they lost excessive amounts of weight, weight loss above the median was not associated with the cessation of exclusive breastfeeding.

## 4 DISCUSSION

There has been an increase globally in the rates of CS, particularly elective CS in recent decades, with 21.1% of all births worldwide being by CS in 2015, almost double the rate reported in 2000 (Boerma et al., 2018). As such, it is important to understand the potential impact of CS on women and their infants in the immediate and longer term post‐natal period and to update guidelines and content and planning of post‐natal care accordingly. The studies included in this review found overall that CS was associated with increased neonatal weight loss in the early days of life compared with infants born vaginally. This is the first systematic review to present these findings, with some caution needed given the small number of relevant studies included, range of data collection methods, and approaches to analysis. Ideally, to carry out an accurate meta‐analysis of the association between neonatal weight loss and mode of birth, we would have known the percentage mean weight loss, statistical significance, and time to weight loss assessment for each study, but weight loss was reported in too many different formats to facilitate this. The use of an integrative review approach enabled a comprehensive review of available literature, much of it from observational data, with a specific focus, and includes data from relevant studies regardless of approaches and methods to data capture. Findings were compared, and ‘gaps' in evidence highlighted.

The studies included were undertaken in a range of countries reflecting high‐income and middle‐income settings and populations; two were based on the same USA cohort, with single studies from Italy, Spain, Iran, India, and Brazil. While it is beneficial to have results from different birth settings, it is not possible to comment on how the model of maternity service provision, population health, and cultural issues may have contributed to infant weight loss. Two papers did not directly compare groups, and although there seemed to be a difference in weight trajectories based on the data presented, they did not provide formal measures of difference (Flaherman et al., 2015; Miller et al., 2015). Of the papers that did report statistical significance, four found that babies born by CS lost more weight within the first 5 post‐natal days (Manganaro et al., 2001; Mezzacappa & Ferreira, 2016; Saki et al., 2010; Samayam et al., 2016), while one found no difference (Verd et al., 2018). Only one study, Saki et al. (2010), examined weight loss separately in male and female infants. Their results suggest that gender may not impact weight change in the first month of life; however, their sample size was too small to reliably assess the impact of gender on neonatal weight loss. There were conflicting results from other studies regarding the effect of gender on weight loss, but these studies did not separate infants based on feeding type and mode of delivery, which may have confounded results (Grossman, Chaudhuri, Feldman‐Winter, & Merewood, 2012; Haseli et al., 2017; Johnen & Daugule, 2018; Regnault et al., 2011).

None of the papers included commented on whether the level of weight loss observed, potentially due to mode of delivery, had been detrimental to the infant's health. Flaherman et al. (2018) did publish another paper on newborn weight loss and health care utilization which found that babies who were born vaginally had similar mean numbers of outpatient visits to those born by CS when divided by level of weight loss (3.7 vs. 3.5 visits for weight loss >10%, 2.9 vs. 2.6 visits for weight loss <8%; Flaherman et al., 2018). However, this paper was excluded from the review as the authors did not differentiate by feeding type, as they did in the other papers presenting data from the same cohort, meaning that we cannot ascertain the health implications of weight linked to mode of delivery (Flaherman et al., 2015; Miller et al., 2015).

The most common reason for papers being excluded was absence of information on feeding type. A recommendation emerging from this review is that authors account for feeding method when reporting infant weight loss based on birth mode, as it can affect weight patterns independent of delivery. Only one paper included formula fed infants, while the six others only included exclusively breastfed infants. It would be interesting to have more studies assessing weight loss in formula fed babies to confirm whether increased weight loss following CS births is mainly restricted to being exclusively breastfed or not; this may be reason to look further into barriers to breastfeeding that may be specific to women who have given birth by CS and give them increased breastfeeding support before weight loss occurs.

Three of the studies included reported percentage mean weight loss of babies born by CS compared with those born vaginally (Flaherman et al., 2015; Miller et al., 2015; Samayam et al., 2016). While all three showed higher mean weight loss in babies born by CS at each timepoint of follow‐up, only Samayam et al. (2016) reported a statistically significant difference. As Miller and Flaherman used the same original cohort, it was interesting to compare weight loss in the formula fed with that of exclusively breastfed groups. Although neither study reported using statistical tests to compare weight loss in vaginally delivered infants with those born by CS, infants born by CS did have slightly higher mean percentage weight loss at 48 hr in Miller et al.'s (2015) formula fed cohort and all timepoints in Flaherman et al.'s (2015) exclusively breastfed cohort. However, mean weight loss was much lower in Miller et al.'s (2015) formula fed cohort than the exclusively breastfed group by Flaherman et al.'s (2015). This is in keeping with other studies in which exclusively breastfed babies had increased weight loss compared with exclusively formula fed babies, which did not take into account the mode of birth (Crossland, Richmond, Hudson, Smith, & Abu‐Harb, 2008; Macdonald, 2003).

Miller et al. (2015) and Flaherman et al. (2015) also reported the proportion of infants who lost excess weight. Miller et al. (2015) found that a similar percentage of infants born by CS or vaginally lost excess weight; however, they defined this as 7% for babies born vaginally and >8% for those born by CS. This is in contrast to Flaherman's findings in the exclusively breastfed cohort, where almost 5% of babies delivered vaginally had lost >10% of birthweight at 48 hr, while almost 10% of CS babies had lost >10% at the same timepoint. Mezzacappa and Ferreira found that birth by CS was associated with an increased risk of excess weight loss with half of babies who lost >8% of birthweight being born by CS while only a quarter of those without excess weight loss born by CS. Similarly, in Manganaro et al.'s study (2001), most infants who lost >10% weight were born by CS, but only a third of babies who lost <10% were born via CS.

Of the papers that compared weight loss by CS or vaginal birth, only Verd et al. (2018) and Manganaro et al. (2001) provided *p*‐values, which were conflicting. Although Verd et al. (2018) did not find a significant association between mode of delivery and weight loss above or below the median, they did separate mode of delivery into “non‐assisted”, “instrumental”, and “caesarean section”. Furthermore, Manganaro et al. (2001) considered association between mode of delivery and weight loss >10%, while Verd et al. (2018) calculated the association between mode of delivery and weight loss above or below the median weight loss of the cohort (6%), which may not be relevant as this is below what is generally considered excess weight loss (Chantry et al., 2011; Davanzo et al., 2013; Mulder & Gardner, 2015). Overall studies indicate that CS has a negative impact on neonatal weight.

While this review mainly focused on the first 5 days of life, two studies recorded weight changes for the first month with conflicting results (Saki et al., 2010; Samayam et al., 2016). Saki et al. (2010) showed that CS infants lost significantly more weight in the early days and gained weight slower than their counterparts born vaginally, although this was rectified by day 25. This suggests that any initial discrepancy in weight loss by mode of birth may be temporary with no long‐term consequences and that intervention may not be necessary. However, Samayam et al. (2016)) report that at day 28, infants born vaginally gained significantly more weight per day than their CS counterparts. Similarly, Mueller, Zhang, Hoyo, Østbye, and Benjamin‐Neelon (2019) recently showed that birth by CS was associated with accelerated weight gain over the first year of life, with divergence appearing as early as 3 months. However, this study did not stratify babies by feeding type (Mueller et al., 2019). Thus, it would be important to increase duration of follow‐up in future studies to determine if weight gain differences seen in the first week of life are remedied after several weeks or months post‐natal.

Weight loss was calculated in four studies around the time of inpatient discharge (Mezzacappa & Ferreira, 2016; Miller et al., 2015; Schaefer et al., 2015; Verd et al., 2018). However, infants born vaginally are often discharged earlier than those born by CS due to maternal recovery time from surgery, introducing possible confounding as vaginally delivered babies may continue to lose weight after discharge. Mezzacappa and Ferreira (2016) accounted for this in their analysis; however, Verd et al. (2018) did not. Miller et al. (2015) and Flaherman et al. (2015) weighed babies every 24 hr until discharge, with an extra timepoint for CS infants due to longer hospital stay (96 hr) but compared weight loss at set timepoints (24 and 72 hr). Similarly, Samayam et al. (2016) and Saki et al. (2010) weighed babies at set timepoints. Manganaro et al. (2001) measured weight loss within 5 days of birth but did not report the exact day weight loss was calculated from. It is clear that the use of set timepoints is essential when researching neonatal weight loss to reduce confounding. This methodological flaw has also been highlighted in other relevant systematic reviews in which authors aimed to establish the reference weight loss of exclusively breastfed infants in the first 2 weeks of life, but due to inconsistencies in the method of reporting weight loss, they were unable to do so (Noel‐Weiss, Courant, & Woodend, 2008; Thulier, 2016).

Another limitation of the included studies is that the type of CS performed was not considered. Preer, Newby, and Philipp (2012) found that exclusively breastfed infants born by pre‐labour CS lost 1.2% more weight than those who were born to women who went into labour (*p* = .004). The absence of labour may affect birthweight and percentage weight loss for a variety of reasons such as delayed clearance of fluid from lungs (Jain & Eaton, 2006), potentially due to lack of “vaginal squeeze”, compression an infant experiences while passing through the birth canal, and fluctuations in hormones (Bland, 2001).

Overall, included studies suggest that babies born by CS lose more weight during the early days of life compared with those born vaginally, although it is unclear if this weight loss is detrimental to the infant as authors did not report rates of hospital readmissions or other adverse outcomes. However, in order to determine whether separate weight loss thresholds should be used for babies born by CS, more studies are need with longer follow‐up periods to assess if discrepancies in weight loss and gain patterns between infants with different modes of birth are remedied several weeks post‐natal. Ideally, this further study should avoid interventions (e.g., encourage topping up with expressed breast milk or donor milk instead of formula feeding) to remedy perceived weight loss based on local practice. It would be useful if studies further explore why infants born by CS might lose more weight, that is, breastfeeding difficulties, delayed lactogenesis, pregnancy problems, and others. Papers should also report neonatal weight loss in a standardised format to allow for comparison. It would be important for studies examining weight loss in babies born by CS to stratify infants by feeding type, exclusively breastfed, formula fed, and mixed feeding, and potentially by emergency or elective CS. Further studies focusing on these considerations are needed to inform policy or guidance and feeding plans for babies born by CS.

### CONFLICTS OF INTEREST

RT is being funded to carry out a randomised controlled trial of probiotic supplementation in babies born by elective caesarean section by Evolve Biosystems (Davis, California), PROMESA – Promotion of a Healthy Gut Microbiome in Elective Caesarean Section Arrivals. Can exclusive breastfeeding supplemented with a probiotic promote a sustained healthy microbiota in babies born by caesarean section? Full details are registered (ISRCTN11690200). NK is employed as a research assistant on this study, while JK and RG were research midwives on this study.

## CONTRIBUTIONS

NK, JK, RG, and RT conceived the research question. NK created the search strategy and conducted the search. NK and RG screened titles and abstracts of the search results. Full texts were screened by NK, RG, and JK. NK created the tables and figures, and along with RG and JK, NK was involved in writing and editing the manuscript, while further writing and editing was completed by RT and DB. PROSPERO: CRD42019125908.
